# Spontaneous Spinal Epidural Hematoma as a Manifestation of Hypertensive Emergency: A Case Report

**DOI:** 10.5811/cpcem.62229

**Published:** 2026-10-13

**Authors:** Kelly Laird, Jordan Sell, Peter England, Sacha Montas, Kevin Walsh, Carrie Harvey

**Affiliations:** * Tacoma Emergency Care Physicians, Tacoma, Washington; † North Memorial Medical Center Emergency Medicine, Robbinsdale, Minnesota; ‡ University of Michigan, Department of Emergency Medicine, Division of Critical Care, Ann Arbor, Michigan; § University of Michigan, Department of Emergency Medicine, Ann Arbor, Michigan

**Keywords:** spontaneous spinal epidural hematoma, hypertensive emergency, case report

## Abstract

**Introduction:**

Uncontrolled hypertension is frequently evaluated in the emergency department (ED), with the goal of identifying those patients with hypertensive emergencies. Spontaneous spinal epidural hematoma is rare and not considered a typical manifestation of hypertensive emergency.

**Case Report:**

A 31-year-old man presenting to the ED with chest pain and hypertension subsequently developed lower extremity neurologic deficits and was diagnosed with a spontaneous spinal epidural hematoma. After thorough testing, no other risk factors for this medical condition were identified, and the hemorrhage was attributed to hypertensive emergency.

**Conclusion:**

Spontaneous spinal epidural hematoma is a rare complication of hypertensive emergency and should be considered when evaluating patients with hypertension, back pain, and neurologic deficits.

## INTRODUCTION

Uncontrolled hypertension, or blood pressure greater than 140/90 mm Hg due to untreated or ineffectively treated hypertension, is frequently evaluated in the emergency department (ED). Hypertensive emergency, defined as an acute and marked elevation in blood pressure with associated target-organ damage, is less common, with a prevalence of 0.5% of ED visits.^1^,^2^ Spontaneous spinal epidural hematoma, or blood in the epidural space in the absence of trauma or other iatrogenic cause, is even more rare, with an estimated incidence of 0.1 in 100,00 per year.^3^ Spontaneous spinal epidural hematoma has been associated with coagulopathy or vascular malformations, but hypertension is not thought to be a risk factor.^3^,^4^ This rare life-threatening neurosurgical emergency can produce devastating neurologic deficits and requires prompt recognition and intervention to reduce morbidity and mortality.^5^ We present a case of a 31-year-old man who developed the medical condition presumed secondary to hypertensive emergency.

## CASE REPORT

A 31-year-old man with a history of hypertension presented to the ED with chest pain. The patient presented with severe central chest pain that began several hours earlier. There was no radiation to the back, although he did report mild back pain that he attributed to his job as a house cleaner. He denied shortness of breath, nausea, vomiting, diaphoresis, headache, neurologic symptoms, or preceding trauma. He had no other medical history but noted he had not been adherent to his antihypertensive medications due to inability to access primary care. Social history was relevant for smoking 0.5 packs per day and weekly marijuana use.

Initial vital signs were notable for marked hypertension with blood pressure (BP) 225/134 mm Hg. Temperature, heart rate, respiratory rate, and oxygenation were normal. On arrival, his physical exam was unremarkable, without cardiac or pulmonary abnormalities noted, and he was able to ambulate without difficulty. It was not immediately clear whether the marked hypertension was the cause or a consequence of his chest pain. Diagnostic testing thus focused on evaluation for serious or life-threatening causes, such as acute coronary syndrome, acute aortic syndrome, pulmonary embolism, pneumothorax, or other end-organ damage from hypertension.

Electrocardiogram showed normal sinus rhythm with likely left ventricular hypertrophy and no other abnormalities. Creatinine was mildly elevated at 1.52 mg/dL (reference range, 0.70–1.30 mg/dL), but complete blood count, prothrombin time, international normalized ratio, partial thromboplastin time and high-sensitivity troponin were all normal. Chest radiograph and computed tomography (CT) of the aorta showed no acute findings or other explanation for his chest pain. There were no abnormal findings within the spinal cord in the final impression or full body of the CT report.

Several hours into his ED course and shortly following CT report finalization, he reported an abrupt onset of numbness to the right lower extremity. Physical examination at that time noted decreased strength to flexion at the right hip and flexion and extension of the right knee and ankle compared to the left, with diminished sensation prompting emergent magnetic resonance imaging (MRI) of the spine with concern for cord compressing process. Over the next 1–2 hours, strength of the right lower extremity decreased, and he developed urinary retention and ascending paralysis into his trunk, although no back pain. Blood pressure was persistently elevated during this time with systolic BP greater than 200 mm Hg. Emergent MRI of his spine noted a posterior thoracic epidural mass at the level of of the first thoracic (T1) to second thoracic (T2) vertebrae measuring 21 mm (Image).

Neurosurgery was consulted due to concern for spontaneous spinal epidural hematoma, and he was taken for urgent open laminectomy with hematoma evacuation. Due to improvement in blood pressure while in the ED during clinical evaluation no antihypertensive medication was provided. Postoperatively, he underwent additional testing to ascertain the etiology of his condition. Spinal angiogram was normal with no arteriovenous malformation or other vascular lesion. Exhaustive workup for underlying coagulopathy was normal, including rotational thromboelastometry, factor XIII, von Willebrand panel, and platelet function testing. The spontaneous spinal epidural hematoma was ultimately ascribed to hypertensive emergency, and he was discharged with near-resolution of his neurologic deficits. Five weeks after surgery, he had no neurologic deficits and had restarted antihypertensive medications.

## DISCUSSION

Spontaneous spinal epidural hematoma is a rare and emergent condition defined as accumulation of blood in the vertebral epidural space without obvious cause. It typically presents with sudden onset of neck or back pain followed by signs of nerve root or spinal cord compression, such as radiculopathy, progressive numbness or weakness, or cauda-equina syndrome.^3^ Symptoms often progress over minutes to hours, although it can be more insidious.^5^ Magnetic resonance imaging is the modality of choice, as it can identify location, extent, and chronicity of the hematoma, as well as presence of cord compression and/or spinal cord edema.^6^

*CPC-EM Capsule*

What do we already know about this clinical entity?

*Spontaneous spinal epidural hematoma (SSEH) is a rare cause of spinal cord or cauda equina nerve compression that occurs in the absence of trauma or other iatrogenic cause.*

What makes this presentation of disease reportable?

*Though SSEH is associated with other risk factors, such as vascular malformations or coagulopathy, it is not a typical manifestation of hypertensive emergencies.*

What is the major learning point?

*Emergency physicians must maintain a high suspicion of this diagnosis when evaluating patients with uncontrolled hypertension, back pain and/or neurologic deficits.*

How might this improve emergency medicine practice?

*Emergency physicians should consider this rare diagnosis to minimize the risk of devastating neurologic sequelae.*

Previously described risk factors for spontaneous spinal epidural hematoma include arteriovenous malformations, anticoagulation use, coagulopathy and hemangiomas, though an estimated 40–60% of cases have no identifiable cause.^3^ Although the condition has been described in patients with hypertension, a recent meta-analysis did not find this to be an independent risk factor.^4^ Additionally, most authors favor epidural veins as the source of bleeding based on anatomy, which would argue against hypertension causing bleeding.^4^,^5^,^7^ However, while preexisting hypertension may not be a risk factor, it is possible that significantly elevated blood pressure may play an unidentified role in development of spontaneous spinal epidural hematoma.^8^,^9^

Urgent surgical evacuation within 12–48 hours is the definitive treatment, with prognosis most dependent on the severity of the preoperative neurologic deficit.^3^–^6^ Unlike other forms of hypertensive emergency, there is no specific treatment guideline regarding blood pressure management. Cheng et al reported aggressive blood pressure lowering in a patient with cervical spontaneous spinal epidural hematoma and marked hypertension, who was treated nonoperatively with complete neurologic recovery.^10^ Small case series in surgical literature have also described increased risk of postoperative spinal epidural hematoma with uncontrolled hypertension (eg, systolic blood pressure > 170 mm Hg.^11,12^ Ultimately, prompt surgical consultation or transfer to an appropriate referral center for definitive management is imperative, with additional medical management of coagulopathy and/or uncontrolled hypertension tailored to the individual patient.

In this case, the patient presented with chest pain and uncontrolled hypertension and underwent a typical ED evaluation for these complaints, which was unrevealing. He then suffered a spontaneous spinal epidural hematoma without any clear precipitating cause or risk factors other than his uncontrolled hypertension. Although emergency physicians frequently evaluate patients for hypertensive emergencies, the focus is typically on select target organs that are more commonly damaged, such as brain, heart, and aorta. Thus, spontaneous spinal epidural hematoma secondary to hypertensive emergency is a rare but important consideration in the differential diagnosis for a patient with uncontrolled hypertension and signs and symptoms of spinal cord compression because prompt recognition and intervention are necessary to minimize permanent neurologic damage.

## CONCLUSION

Uncontrolled hypertension can lead to highly morbid conditions and is frequently evaluated in the ED. Spontaneous spinal epidural hematoma is a rare complication of hypertensive emergency and should be considered in the emergency evaluation of a patient with uncontrolled hypertension in addition to typical symptoms and signs of spinal cord compression or cauda-equina syndrome.

## Figures and Tables

**Image:**
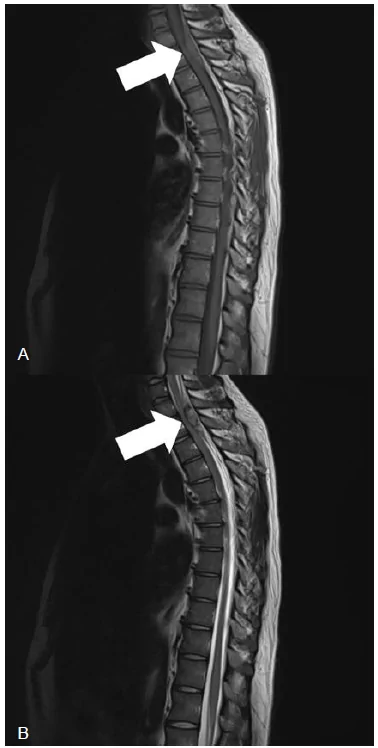
Sagittal magnetic resonance imaging of the thoracic spine demonstrating a 21-mm posteriorly positioned mass within the thoracic spinal canal at the level of first and second thoracic vertebrae, which results in severe thecal sac narrowing and subtle cord signal alteration at the level of first thoracic. The mass does not demonstrate contrast enhancement on T1-weighted images (A) with peripheral and internal curvilinear hypointensity on T2-weighted images (B), which is most suggestive of epidural hematoma.
